# Frequency Up-Conversion Photon-Type Terahertz Imager

**DOI:** 10.1038/srep25383

**Published:** 2016-05-05

**Authors:** Z. L. Fu, L. L. Gu, X. G. Guo, Z. Y. Tan, W. J. Wan, T. Zhou, D. X. Shao, R. Zhang, J. C. Cao

**Affiliations:** 1 Key Laboratory of Terahertz Solid-State Technology, Shanghai Institute of Microsystem and Information Technology, Chinese Academy of Sciences, 865 Changning Road, Shanghai 200050, China

## Abstract

Terahertz imaging has many important potential applications. Due to the failure of Si readout integrated circuits (ROICs) and the thermal mismatch between the photo-detector arrays and the ROICs at temperatures below 40 K, there are big technical challenges to construct terahertz photo-type focal plane arrays. In this work, we report pixel-less photo-type terahertz imagers based on the frequency up-conversion technique. The devices are composed of terahertz quantum-well photo-detectors (QWPs) and near-infrared (NIR) light emitting diodes (LEDs) which are grown in sequence on the same substrates using molecular beam epitaxy. In such an integrated QWP-LED device, photocurrent in the QWP drives the LED to emit NIR light. By optimizing the structural parameters of the QWP-LED, the QWP part and the LED part both work well. The maximum values of the internal and external energy up-conversion efficiencies are around 20% and 0.5%. A laser spot of a homemade terahertz quantum cascade laser is imaged by the QWP-LED together with a commercial Si camera. The pixel-less imaging results show that the image blurring induced by the transverse spreading of photocurrent is negligible. The demonstrated pixel-less imaging opens a new way to realize high performance terahertz imaging devices.

Terahertz imaging has many potential applications, such as deep space exploration, imaging of biological tissues, mail screening and fingerprint identification, and situation awareness in fire disasters^1^. Terahertz focal plane arrays (FPAs) are key components for real time terahertz imaging systems^2^. Several different FPAs working in the terahertz regime have been developed in recent years based on the phase change of VO_x_, the complementary metal-oxide-semiconductor transistor (CMOS) technology, and the pyroelectric effect of LiTaO_3_ crystal^2^,^3^. However, photo-type terahertz FPAs are still absent now. Compared to the thermal and other FPAs, photo-type FPAs have advantages of high detection sensitivity, short response time, large linear response range, and high damage threshold^2^.

In mid- and far-infrared spectral regimes, FPAs are constructed by flip-chip bonding photo-detector arrays to commercial Si readout integrated circuits (ROICs)^2^. HgCdTe and quantum well infrared photo-detector (QWIP) FPAs are in the dominant position in mid- and far-infrared regimes^4^. Terahertz photo-detectors based on inter-subband transition in one- and three-dimensional quantum confined semiconductor structures were demonstrated in the past decade^2^,^4^. GaAs/(Al,Ga)As quantum well photo-detectors (QWPs) operating in terahertz band were realized by Liu *et al.*^5^. At temperatures below 30 K, background-limited infrared performance (BLIP) is reached for terahertz QWPs in 3.0–7.0 THz^6^. Due to the high quality of molecular beam epitaxy (MBE) grown GaAs/(Al,Ga)As multi-layer structure and the mature III-V fabrication processing technique, it is possible to fabricate terahertz QWPs with uniform detection performance in a large area scale. However, there are two technical difficulties to fabricate hybrid terahertz QWP FPAs by flip-chip bonding the terahertz QWPs to the Si ROICs. First, terahertz QWPs must be operated at temperatures lower than 30 K to suppress dark current. A high-level dark current will seriously degrade the imaging performance due to the saturation of readout capacitors. Below the temperature of 40 K, commercial Si ROICs will lose their functions^2^. It is a very resource-demanding target to design and fabricate the Si ROICs operated at very low temperatures. Secondly, because of thermal mismatch between GaAs/(Al,Ga)As and Si, the Indium bumps for electrical connection between the photo-detector elements and the ROIC cells will be destroyed, and the number of dead pixels will increase with decreasing temperature.

Liu *et al.* have developed the frequency up-conversion technique by integrating a QWIP and a light emitting diode (LED)^7^. When the QWIP-LED is illuminated by mid- or far-infrared radiation, the resistance change of the QWIP will result in a change of near-infrared (NIR) emission intensity of the LED. Therefore, the serially integrated QWIP-LED device is a frequency up-converter, in which the NIR emission intensity is modulated by the mid- or far-infrared radiation shed on the device. The subsequent theoretical and experimental investigations on the QWIP-LED devices show that the lateral spreading of photocurrent is negligible^8^,^9^,^10^,^11^,^12^,^13^; large area pixel-less FPAs based on QWIP-LEDs without obvious picture smearing and distortion effects were demonstrated by using a Si charge-coupled device (CCD) or CMOS camera to detect the converted NIR emission pattern^10^,^11^,^12^,^13^.

It is technically important to explore pixel-less imaging using a frequency up-conversion device together with a NIR camera mentioned above in terahertz regime due to the absence of commercial Si ROICs working below 40 K^2^. In this paper, we design and fabricate terahertz QWP-LED frequency up-conversion devices that can effectively convert terahertz radiation into NIR emission. Despite the large difference of the bias voltages between terahertz QWPs (~0.2 V) and LEDs (~1.3 V for GaAs/(In,Ga)As material system), the optimized terahertz QWP-LED devices work well at forward bias voltages of 1.45–1.65 V and in the temperature range of 3.5–10 K. In such bias and temperature operation conditions, the ratio of photocurrent and background current can reach 100, and the internal quantum efficiency (IQE) of light emission in the LED part is larger than 90%. The energy conversion efficiency is about 4 × 10^−4^, which is severely limited by the poor light extraction efficiency (LEF) of around 2.5%. The laser spot of a homemade terahertz quantum cascade laser (QCL) is imaged by the terahertz QWP-LED together with an Andor Si CCD camera (iKon-M 934 BR-DD). The demonstrated frequency up-conversion pixel-less imaging technique opens a new way to realize high performance photo-type terahertz imaging devices.

## Device structure and electrical characterization

**Figure 1:**
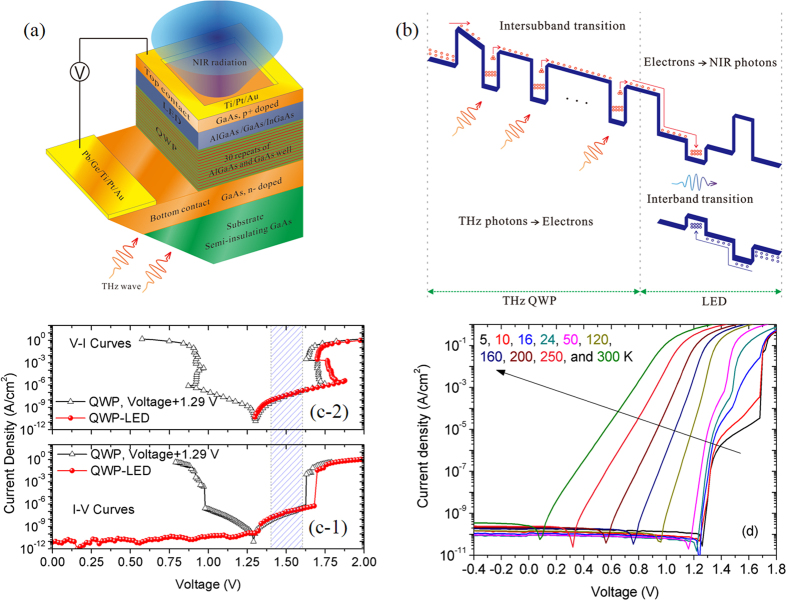
(**a**) The schematic of the GaAs/(Al,Ga)As/(In,Ga)As 45-degree facet coupled terahertz QWP-LED; (**b**) Band-edge profiles and operation principle of QWP-LEDs; (**c**) Dark (4.2 K) and 300 K background current-voltage (I-V) curves of the QWP-LED and a comparison terahertz QWP having the same MQW parameters with the QWP part of the QWP-LED. The current-voltage curves of the QWP are shifted along the positive *x* axis by 1.29 V for convenience; (**d**) The background current-voltage curves at different temperatures.

The terahertz QWP-LED devices are based on the GaAs/(Al,Ga)As/(In,Ga)As material system. The multi-quantum well (MQW) structural parameters are optimized by solving the coupled Schrodinger and Possion equations. The exchange-correlation and depolarization many-particle effects are considered within the local density approximation^14^. The device structures were grown by MBE on 600 μm thick semi-insulating GaAs substrates. As shown in Fig. 1(a), the device is composed of a GaAs/(Al,Ga)As QWP and an (In,Ga)As/GaAs LED (from the substrate). The QWP structure consists of a 800 nm thick bottom contact layer doped with Si to 1.0 × 10^17^/cm^3^, a 80 nm Al_0.024_Ga_0.976_As emission barrier, and 30 repeats of GaAs/Al_0.024_Ga_0.976_As MQWs with barrier and well widths of 80 nm and 16 nm, and 1.0 × 10^17^/cm^3^ Si doping in the central 10 nm region of each well. The LED structure consists of a 40 nm thick GaAs layer, a 9 nm thick In_0.1_Ga_0.9_As layer, a 40 nm thick GaAs layer, and an 140 nm thick Al_0.024_Ga_0.976_As confined layer with Be doping to 5.0 × 10^18^/cm^3^, and a 50 nm thick GaAs top contact layer doped with Be to 8.0 × 10^18^/cm^3^. Square mesa structures with 1.0 mm edge length were fabricated using optical lithography and wet-chemical etching. The top electrical connections were narrow Ti/Pt/Au ring contacts. The bottom contact was made of Pb/Ge/Ti/Pt/Au. To fulfill the inter-subband transition selection rule, a 45-degree facet near the mesa was mechanically polished, through which terahertz radiation is coupled into the mesa. Figure 1(b) depicts the conduction and valance band edge profiles and the operation principle of terahertz QWP-LEDs. There are two advantages to adopt a thin In_0.1_Ga_0.9_As layer as the active radiation recombination region. First, in steady state and in the condition of fixed injection current, the electron and hole densities in the radiation recombination layer increase with decreasing In_0.1_Ga_0.9_As layer thickness, which is benefit for acquiring high emission internal quantum efficiency (IQE)^15^, especially in the case of small injection current. Secondly, due to the narrower band-gap of In_0.1_Ga_0.9_As, the emitted photons from the In_0.1_Ga_0.9_As layer cannot be absorbed severely by GaAs matrix, which results in higher energy conversion efficiencies of terahertz QWP-LEDs. Moreover, at low temperatures, the frequency of photons emitted by In_0.1_Ga_0.9_As is very close to the peak response frequency of Si photon diodes.

The main application scenario of terahertz QWP-LEDs is pixel-less terahertz imaging, which determines that no highly conductive layer can be inserted between the QWP part and the LED part of a QWP-LED because the electron-electron scattering in such a conductive layer will strongly smear the target picture^13^. Therefore, the terahertz QWP-LEDs for pixel-less FPAs must be designed as two-terminal devices, which means that we cannot adjust the values of bias voltage applied to the QWP part and the LED part separately. The dark current-voltage (I-V, voltage sweeping) and voltage-current (V-I, current sweeping) curves of a QWP-LED and a test QWP with the same MQW structure are measured using a source-measure unit (Keithley Model 238) at 4.2 K. In order to screen the uncontrollable influences from environment, the devices under measurement are immerged into liquid Helium. Figure 1(c) shows the I-V and V-I curves of the terahertz QWP-LED and the single terahertz QWP. There are big current jumps both in the I-V curves of the QWP-LED and the test QWP. The corresponding negative differential resistance regions are also found both in the V-I curves^16^,^17^. After shifting the bias voltage of the terahertz QWP by 1.29 V, we find that the positive branches of I-V and V-I curves of the test QWP and the QWP-LED are almost in superposition with each other at the bias voltage range of 1.45–1.65 V, which is the optimal operation bias window for the terahertz QWP-LED. In this bias window, the electrical field distribution in the QWP part is not evidently distorted by the LED part. The measurement results shown in Fig. 1(c) also indicate that the built-in voltage of the LED part is about 1.29 V, and the junction resistance of the LED part is negligible when the bias voltage is lower than 1.70 V. The background I-V curves at different temperatures are displayed in Fig. 1(d). At temperatures of lower than 50 K, the features of the I-V curves are dominated by the QWP part when the bias voltage is higher than the forward threshold voltage of the LED part. With the further increase of temperature, because the QWP part becomes a pure resistor and the resistance decreases, the I-V curves of the QWP-LED are similar with those of traditional LEDs^15^. The electrical characteristics shown in Fig. 1(c,d) indicate that we can find an optimal bias applied to the QWP-LED to make the QWP part and the LED part both in their optimal operation conditions.

## Terahertz photo-response and NIR emission

**Figure 2:**
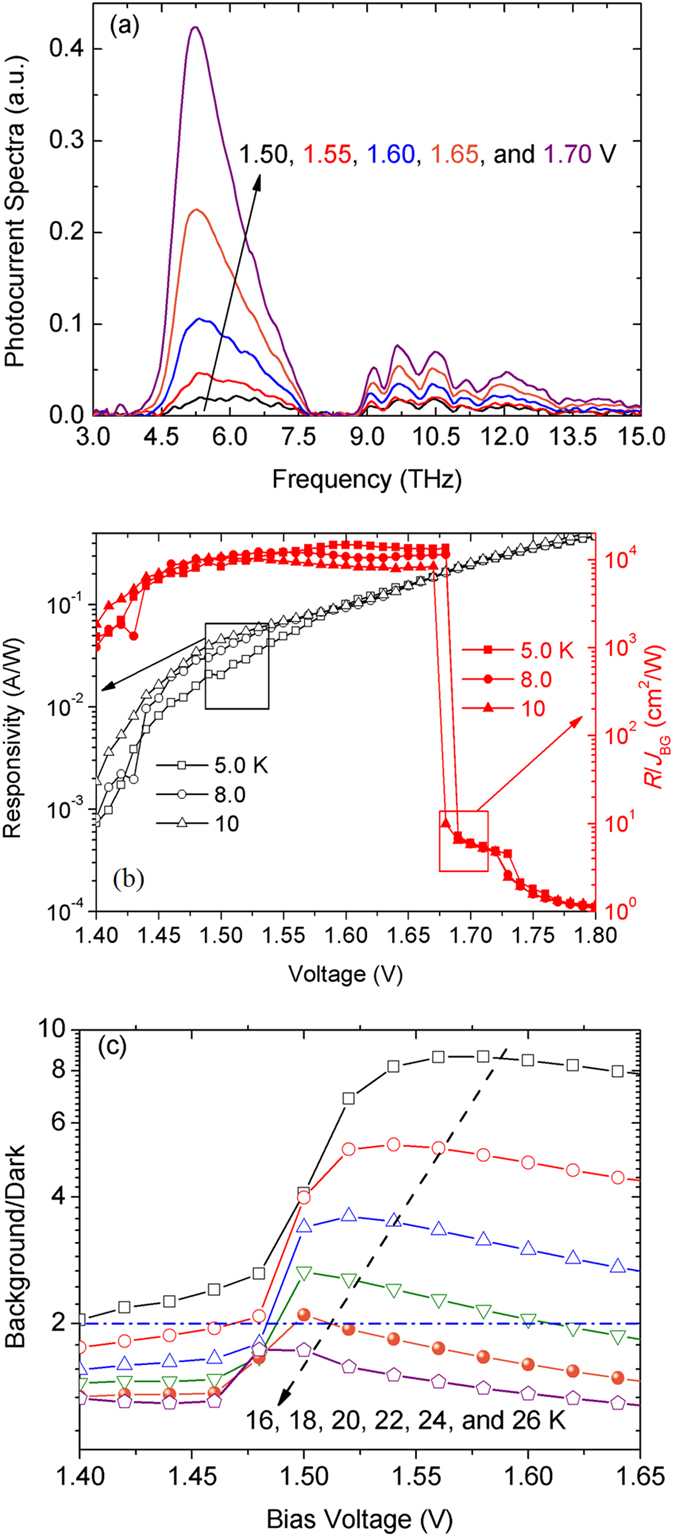
(**a**) Photocurrent spectra of the QWP-LED at 5.0 K and at different bias voltages; (**b**) Responsivities and the values of *R*/*J*_BG_ of the QWP-LED at 5.0 K and at different bias voltages; (**c**) *J*_BG_/*J*_Dark_ – voltage relations at different temperatures for derivation of BLIP temperature.

The photocurrent spectra at 5.0 K and different bias voltages shown in Fig. 2(a) are measured by using a Fourier transform infrared spectrometer (Bruker IFS 66 v/s). With the increase of bias voltage, the peak position shifts to lower frequency and the peak magnitude raise exponentially, which is a typical characteristic of bound-bound inter-subband transitions^18^. The microscopic mechanism is that a higher bias voltage make the effective energy barriers become lower and narrower, and the photo-excited electrons in the second subband have more probability to escape from the quantum well. The linear increase of photocurrent in the range of 9.0–15.0 THz is due to the reduction of electron transit time brought by the increase of bias voltage. There is a discrepancy of peak response frequency between the calculated one (4.5 THz) and the measured one (5.2 THz). Such a discrepancy maybe originates in the excess of Al fraction in barriers or the underestimate of many-particle effects. Further investigations are needed to address the exact reason of this discrepancy.

The peak responsivities of the QWP-LED at different bias voltages shown in Fig. 2(b) are acquired using a calibrated blackbody (Infrared Systems Development Corporation IR-564/301). The peak responsivity is about 0.22 A/W at bias of 1.67 V and at temperature range of 5.0–10 K. We define a quantity *R*/*J*_BG_ with *R* the responsivity and *J*_BG_ the background current density, to describe the imaging performance of the QWP-LED. The quantity *R*/*J*_BG_ is the ratio of the produced signal current to the background current per one-watt radiation at the peak response frequency of the QWP-LED impinging on the one-cm^2^ device. As displayed in Fig. 2(b), the maximum value of *R*/*J*_BG_ is 1.3 × 10^4 ^cm^2^/W, which implies that the ratio of signal/background currents is 1.0 when the power of radiation at 5.2 THz impinging on the one-cm^2^ QWP-LED is 76.9 μW. The big jump in the I-V curves presented in Fig. 1(c,d) is responsible for the steep decrease of *R*/*J*_BG_ for *V* > 1.68 V.

In order to derive the background-limited infrared performance (BLIP) temperature, the ratio of 300-K background current and the dark current at the bias voltages of 1.40–1.65 V and at different operation temperatures is presented in Fig. 2(c). The BLIP temperature is about 24 K. The measured results of the photocurrent spectra, the values of responsivity, and the BLIP temperature of the terahertz QWP-LED are all similar with those of traditional terahertz QWPs^4^. Therefore, all these experimental results indicate that there are no noticeable negative effects of the LED part on the performance of the QWP part in the QWP-LED.

**Figure 3:**
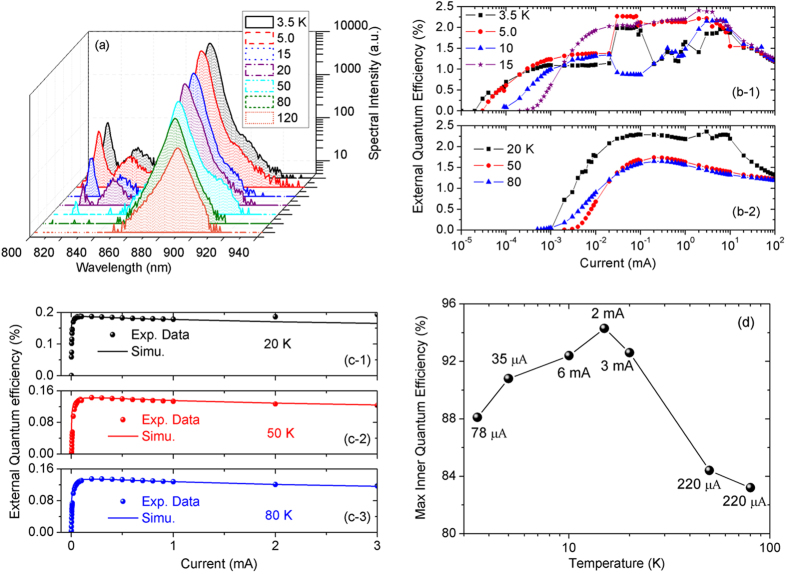
(**a**) Emission spectra of the QWP-LED at a drive current of 10 μA and at different temperatures; (**b**) External quantum efficiencies of the QWP-LED at different temperatures; (**c**) Simulation of external quantum efficiencies based on the ABC model with the maximum internal quantum efficiencies as fitting parameters; (**d**) The maximum values of the internal quantum efficiencies at different temperatures.

The light emission spectra, the internal quantum efficiency, and the light extraction efficiency of the LED part are presented in Fig. 3. As shown in Fig. 3(a), the emission spectra at a drive current of 10 μA and at different temperatures are measured using a portable fiber spectrometer (Ocean optics QE65PRO) with the same setting parameters. The amplitude of the main emission peaks in the range of 878–889 nm decreases sharply with increasing temperature. The red-shift of the main emission peak with increasing temperature is due to the temperature-induced band-gap shrink^19^. As the temperature is decreased below 20 K, there are two extra emission peaks at 814 nm and 833 nm, respectively. In order to clarify the origins of the emission peaks, the band structures of the (Al,Ga)As/GaAs/(In,Ga)As QW are calculated by solving the Schrodinger equation. The band-gaps of GaAs, Al_0.024_Ga_0.976_As, and In_0.1_Ga_0.9_As are taken as 1.520, 1.562, and 1.367 eV, respectively^19^. The ratio of conduction- and valence-band-offsets is assumed to be 6:4. The effective electron (hole) masses for Al_0.024_Ga_0.976_As, GaAs, and In_0.1_Ga_0.9_As are 0.068 (0.57) m_0_, 0.067 (0.56) m_0_, and 0.062 (0.53) m_0_ with m_0_ the electron mass, respectively. Compared to the calculated data, we identify that at temperature of 5.0 K, the peaks at 814 nm, 833 nm, and 878 nm are due to the transitions of ground electron-hole subbands in the wide GaAs QW (814.2 nm, calculated transition wavelength), the second electron-hole subbands in the In_0.1_Ga_0.9_As QW (839.5 nm), and the ground electron-hole subbands in the In_0.1_Ga_0.9_As QW (886.3 nm), respectively. The discrepancies between the experiment and calculation related to the transitions in the In_0.1_Ga_0.9_As QW are due to the neglects of Stark shift induced by the bias and the effect of strain field originating from the lattice mismatch between GaAs and In_0.1_Ga_0.9_As. As the temperature is increased (>20 K), the disappearances of the peaks at 814 nm and 833 nm are attributed to the increase of non-radiation recombination probability, the decrease of electron (hole) mean free path, and the increase of electron (hole) relaxation rate to the ground subbands in In_0.1_Ga_0.9_As QW due to LO-phonon scattering.

As presented in Fig. 3(b), the external quantum efficiencies (EQEs) of the QWP-LED as a function of forward drive current at different temperatures are measured. At drive currents of lower than 2.0 × 10^−4 ^mA, the value of EQE increases with decreasing temperature, which indicates that the non-radiation transition is suppressed at low temperatures, and at such low temperatures, the doped Be acceptors are effectively activated. At the operation temperature of lower than 15 K, the EQE-current curves show complicated behaviors when the drive current is in the range of 2.0 × 10^−2^−10.0 mA (Fig. 3(b-1)). We attribute such anomalous behaviors to the complicated transport properties of electrons induced by the QWP part and the electrons in the wide GaAs QW cannot be effectively captured by the In_0.1_Ga_0.9_As QW. When the working temperature is higher than 20 K, as depicted in Fig. 3(b-2), the anomalous behaviors in the EQE-current curves disappear, and the ABC model can describe the relation of EQE and drive current. By numerically solving the Equation^20^


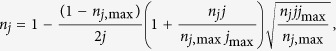


where 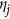 is the IQE, 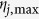 is the maximum value of IQE, *J* is the drive current, and *J*_*max*_ is the drive current at which the maximum value of EQE is achieved. The experimental EQE-current curves at temperatures of 20, 50, and 80 K are fitted with 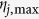 as the fitting parameter. At moderate drive currents, the experimental EQE-current curves are well reproduced by the ABC model^20^. From the data of EQE and IQE, the LEE value of about 2.5% is derived. By assuming that the LEF is insensitive to temperature (a constant at different temperatures)^15^, the values of 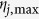 at different operation temperatures are acquired (Fig. 3(d)). At temperature of 5.0 K and at forward bias of 1.65 V, the maximum value of the external frequency up-conversion energy efficiency is 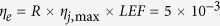.

In this work, we use a terahertz QCL as the signal radiation source^21^. Since the QCL-induced photocurrent is in the range of 1–100 μA, we select an operation point at temperatures of 3.5–8.0 K, at which the IQE of the LED part is higher than 90%. To detect weaker terahertz radiation, further investigations are needed to improve the IQE of the LED part at very low drive currents.

## Frequency up-conversion pixel-less imaging

**Figure 4:**
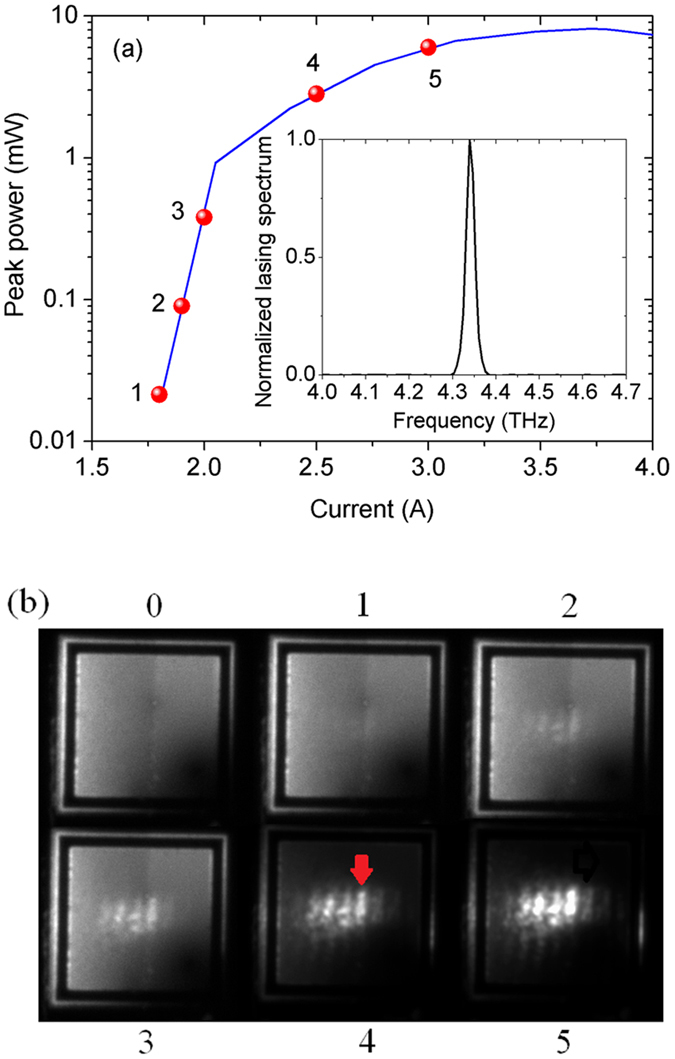
(**a**) Peak-power-current relation of the terahertz QCL and the lasing spectrum (insertion) at 10 K; (**b**) The focal laser spots of the terahertz QCL at 10 K and at different drive currents imaged by the QWP-LED at bias voltage of 1.65 V and at 10 K.

A focal laser spot of a homemade terahertz QCL is imaged by the QWP-LED to check the imaging performance of the device. The structural parameters of the QCL were reported in ref. 21. Figure 4(a) represents the lasing spectrum and the emission peak power as a function of drive current of the QCL at temperature of 10.0 K. The QCL is operated in pulse mode with the repetition frequency of 2.0 kHz and the duty cycle of 1%. The lasing frequency is 4.34 THz, and the maximum peak power is 8.1 mW (averaged power of 81 μW measured by a calibrated thermal detector). The lasing frequency (4.34 THz) of the QCL does not match with the peak response frequency (5.2 THz) of the QWP-LED. The responsivity of the QWP-LED at 4.34 THz (10.5 mA/W) is only about 7.0% of that at the peak response frequency of 5.2 THz (0.22 A/W) at working temperature of 5.0 K and the drive bias voltage of 1.65 V. The lasing beam from the terahertz QCL is collimated and focused onto the 45-degree facet of the QWP-LED perpendicular to the optical axis by two off-axis parabolic mirrors with the same parameters. The peak photocurrents of the QWP-LED are 0.23, 0.90, 3.5, 28, and 50 μA illuminated by the QCL at drive currents of 1.8, 1.9, 2.0, 2.5, and 3.0 A, respectively. The corresponding calculated (*R* × peak power of the QCL) peak photocurrents are 0.23, 0.98, 4.1, 31, and 65 μA, respectively. The deviations between the calculated and the measured peak photocurrents are due to the absorption and reflection of the laser beam energy by the polythene window.

As shown in Fig. 4(b), the terahertz laser focal spots having different powers are imaged by the QWP-LED at the bias of 1.65 V. An Andor Si CCD camera is used to image the emission pattern of the QWP-LED. The exposure time and the integration time of the camera are 1.0 second and 5.0 seconds. Figure 4(b-0) represents the emission pattern of the QWP-LED drive by the background current with the QCL in the off-state. The emission non-homogeneity of the QWP-LED is attributed to the in-plane non-uniformity of the MBE-grown multi-layer structure. Such emission non-homogeneity becomes stronger with decreasing temperature. For the case of 2.3 nA averaged photocurrent (laser spot peak power of 21 μW), it starts to resolve the laser spot in the emission plane of the QWP-LED (Fig. 4(b-1)). When the averaged photocurrent is larger than 9.0 nA (laser spot peak power > 90 μW), the laser spot is well resolved and it becomes clearer with increasing the laser spot power. A dynamic range of about 26 dB in imaging for the QWP-LED is reached. Since the laser spot is projected on the 45-degree facet of the QWP-LED, the imaging of the laser spot is elongated by a factor of 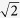 in the horizontal direction. Due to the poor off axis imaging performance of off-axis parabolic mirrors and non-ideal alignment of the optical path, there is an obvious distortion of the imaged spot in the vertical direction. The dimension of the emission facet of the QCL is 10 × 240 μm^2^. Considering the radiation wavelength of 69.1 μm, the QCL is regarded as a linear light source. Therefore, there are parallel Fraunhofer diffraction fringes in the imaging of the laser spot. In Fig. 4(b-5), the bright stripes in the images of the laser spot can be clearly resolved. The width of the fringe with less distortion marked by the red arrow in Fig. 4(b-4) is about 50.0 μm (0.72 λ), which is close to the diffraction limitation, 0.61 λ. The length of the above fringe is about 250 μm that is larger than the real ridge width of 240 μm of the QCL. The imaging spatial resolution indicates that there are no notable transverse spreading of photocurrent in the QWP-LED.

## Discussion

For real applications, the NIR emission plane of terahertz QWP-LEDs should be enlarged. When the projected terahertz pictures fully filling to the 45-degree facet along the 45-degree direction cannot cover the whole emission area of the QWP-LEDs, there exist a dead region in the NIR emission plane. Therefore, instead of the 45-degree facet coupling, metal-grating and other periodic metal structures are better choices for the coupling of terahertz radiation into the QWP-LEDs^22^,^23^. Because the dimensions of the metal structures are smaller than or comparable to the wavelength of terahertz radiation, they are expected to have no negative effects on the pixel-less imaging performance. Meanwhile, some optimized metal structure can provide perfect energy-feeding into the MQW absorption layer^22^,^23^, which will essentially improve the performance of QWP-LEDs.

Noise equivalent power (NEP) is a figure of merit for photodetectors^4^. To evaluate the detection sensitivity of QWP-LEDs, a Si photodiode and a QWP-LED are utilized to construct a detection system. Only the generation-recombination (GR) noises in the QWP part of the QWP-LED and the Si photodiode are considered^4^. For the Si photodiode, there are two noise sources 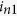 and 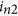 corresponding to the fluctuation of NIR emission from the QWP-LED and the NIR 300-K background radiation, respectively.


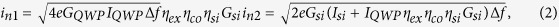


where *e* is the electron charge, 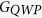 and 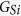 are the photoconductive gains of the QWP and the Si photodiode, 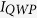 and 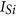 are the 300-K background currents, 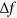 is the measurement bandwidth, 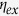 is the EQE of the LED, 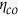 is the collection efficiency, and 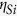 is the absorption quantum efficiency of the Si photodiode, respectively. The total noise current is


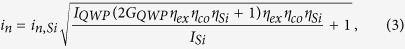


where 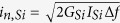 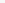
is the GR noise current of the Si photodiode. The signal current in the Si photodiode 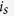 is


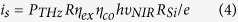


where 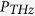 is the power of incident terahertz radiation, *h* is the Plank’s constant, 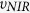 is the frequency of NIR emission from the QWP-LED, and 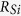 is the responsivity of the Si photodiode, respectively. We obtain the NEP of the up-conversion detective system with 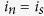,


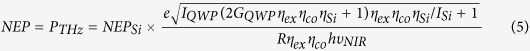


where 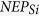 is the NEP of the Si photodiode. If we set 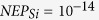
W. 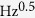, 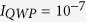
A, 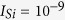 A, 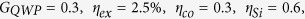 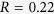
A/W, and 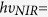
1.4 eV, the NEP of the QWP part is 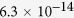 W. 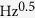, and the NEP of the up-conversion system is 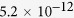 W. 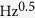. From Eq. (5), we find that the EQE of NIR emission and the collection efficiency of Si photodiodes or Si cameras are key factors to improve the sensitivity of the QWP-LEDs. The micro-cavity-enhanced LED^15^,^24^ and the efficient coupling between the QWP-LEDs and Si photo-detectors/cameras will be investigated further to improve the performance of the QWP-LEDs.

## Conclusions

In this paper, we designed and fabricated frequency up-converters, GaAs/(Al,Ga)As QWP-LEDs working in terahertz regime by integrating QWPs and LEDs on the same GaAs substrates in series. Electrical characteristics showed that the QWP part and the LED part in a QWP-LED can both reach their optimal operation conditions at the biases of 1.45–1.65 V and at the temperatures of 3.5–10 K. The measured results of photocurrent spectra, responsivities, and BLIP temperature of the QWP part and the IQE of the LED part further indicate that there is no appreciable negative interruption between the two parts in the QWP-LED. The pixel-less terahertz imaging was demonstrated using the QWP-LED together with a commercial Si camera. The pixel-less imaging spatial resolution is close to the diffraction limitation, which implies that the transverse spreading of photocurrent is negligible. Performance improvements of the QWP-LEDs, including the applications of grating-coupler with higher terahertz coupling efficiency and the micro-cavity to enhance the LEF of LED, were discussed. There is a big room to improve the performance of the terahertz QWP-LEDs. The demonstrated pixel-less imaging technique provides a new solution to realize high performance terahertz imaging devices.

## Additional Information

**How to cite this article**: Fu, Z. L. *et al.* Frequency Up-Conversion Photon-Type Terahertz Imager. *Sci. Rep.* **6**, 25383; doi: 10.1038/srep25383 (2016).
